# Molecular Characterization of Notch1 Positive Progenitor Cells in the Developing Retina

**DOI:** 10.1371/journal.pone.0131054

**Published:** 2015-06-19

**Authors:** Galina Dvoriantchikova, Isabel Perea-Martinez, Steve Pappas, Ariel Faye Barry, Dagmara Danek, Xenia Dvoriantchikova, Daniel Pelaez, Dmitry Ivanov

**Affiliations:** 1 Bascom Palmer Eye Institute, Department of Ophthalmology, University of Miami Miller School of Medicine, Miami, Florida, United States of America; 2 Department of Biomedical Engineering, University of Miami, Coral Gables, Florida, United States of America; 3 Department of Microbiology and Immunology, University of Miami Miller School of Medicine, Miami, Florida, United States of America; Universidade Federal do ABC, BRAZIL

## Abstract

The oscillatory expression of Notch signaling in neural progenitors suggests that both repressors and activators of neural fate specification are expressed in the same progenitors. Since Notch1 regulates photoreceptor differentiation and contributes (together with Notch3) to ganglion cell fate specification, we hypothesized that genes encoding photoreceptor and ganglion cell fate activators would be highly expressed in Notch1 receptor-bearing (Notch1^+^) progenitors, directing these cells to differentiate into photoreceptors or into ganglion cells when Notch1 activity is diminished. To identify these genes, we used microarray analysis to study expression profiles of whole retinas and isolated from them Notch1^+^ cells at embryonic day 14 (E14) and postnatal day 0 (P0). To isolate Notch1^+^ cells, we utilized immunomagnetic cell separation. We also used Notch3 knockout (Notch3KO) animals to evaluate the contribution of Notch3 signaling in ganglion cell differentiation. Hierarchical clustering of 6,301 differentially expressed genes showed that Notch1^+^ cells grouped near the same developmental stage retina cluster. At E14, we found higher expression of repressors (Notch1, Hes5) and activators (Dll3, Atoh7, Otx2) of neuronal differentiation in Notch1^+^ cells compared to whole retinal cell populations. At P0, Notch1, Hes5, and Dll1 expression was significantly higher in Notch1^+^ cells than in whole retinas. Otx2 expression was more than thirty times higher than Atoh7 expression in Notch1^+^ cells at P0. We also observed that retinas of wild type animals had only 14% (P < 0.05) more ganglion cells compared to Notch3KO mice. Since this number is relatively small and Notch1 has been shown to contribute to ganglion cell fate specification, we suggested that Notch1 signaling may play a more significant role in RGC development than the Notch3 signaling cascade. Finally, our findings suggest that Notch1^+^ progenitors—since they heavily express both pro-ganglion cell (Atoh7) and pro-photoreceptor cell (Otx2) activators—can differentiate into either ganglion cells or photoreceptors.

## Introduction

The number of people suffering from retinal diseases is expected to increase significantly over the next two decades, especially as the population ages [1–3]. These diseases lead to retinal damage and, ultimately, blindness [1–3]. Unfortunately, many retinal diseases currently remain difficult or impossible to treat [1–3]. Stem cell technology harbors a unique potential to solve these treatment conundrums and restore patient vision by repairing and/or regenerating damaged retinas [4–7]. However, efficient use of this technology will require a deeper understanding of the molecular mechanisms of retinal neurogenesis. Although significant progress has been made in this field over the past twenty years [8–10], many important questions remain unanswered; in particular, serious attention must be devoted to reconstructing the gene networks that regulate retinal development.

The retina is generated from multipotent progenitor cells that give rise to ganglion cells, amacrine cells, horizontal cells, and cone photoreceptors in the early stages of retinal development, and to rod photoreceptors, bipolar cells, and Müller glia in the late stages of retinal development [8–10]. A continuous supply of retinal progenitor cells (RPCs) is required for the steady production of differentiated neurons and complete retinal development [8–10]. The Notch pathway is an evolutionarily conserved intercellular signaling cascade that prevents differentiation of RPCs into retinal neurons and facilitates RPC proliferation, thereby maintaining a population of undifferentiated RPCs in the developing retinal tissue [8–11]. The classical view of Notch signaling states that in order to prevent differentiation of progenitors into neurons, the Notch receptor has to be activated by a Notch ligand from an adjacent cell [8–11]. Notch activation triggers release and translocation into the nucleus of the Notch protein’s intracellular domain (ICD) [8–11]. In the nucleus, the ICD binds to the Rbpj transcription factor and activates members of the Hes (hairy and enhancer of split) family, such as Hes1 and Hes5 [8–11]. These proteins repress expression of pro-neural transcription factors (activators of neural fate specification) in progenitors, effectively precluding neuronal differentiation [8–11]. Congruently, reduced Notch activation (and the concomitant reduced inhibitory influences of Hes1 and Hes5) permits neuronal-specific gene expression and neuronal differentiation [8–11]. It has become clear more recently, however, that this classical model inadequately describes certain nuances of Notch signaling. Real-time imaging analysis indicates that Notch signaling in progenitor cells is not static, as previously thought, but dynamic (oscillatory) [12–17]. Notch signaling promotes cyclic expression of both repressors and activators of pro-neural fate specification in progenitor cells [12–17]. As a result, the expression of both repressors and activators of pro-neural fate specification was observed in progenitor cells including in RPCs [12–20]. Meanwhile, pro-neural activators are expressed continuously in post-mitotic neurons in which the inhibitory activity of Notch signaling is diminished [12–17].

There are four Notch receptors (Notch1, Notch2, Notch3, and Notch4), all of which play different roles in retinal development. Notch1 and Notch3 are involved in retinal neurogenesis, while Notch4 is primarily expressed in the vasculature, regulating vessel growth in the retina [21–25]. Notch2 expression appears the least important, and is only slightly detectable in developing retinas [26, 27]. Among these four Notch receptors, Notch1 plays the most important role in the developing retina, as Notch1 is essential for the maintenance of an RPC population [8, 21–24]. Notch1 is also required for inhibiting RPC differentiation into cone (early stage) and rod (late stage of retinal differentiation) photoreceptors, since reduction of Notch1 activity in early RPCs leads to cone photoreceptor production and ablation of Notch1 in late RPCs facilitates rod photoreceptor production [8, 21–24]. It has also been demonstrated that Notch1 (as well as Notch3) regulates ganglion cell fate specification in the developing retina [8, 21]. However, the decision-making mechanism that induces Notch1 positive (Notch1^+^) RPCs to differentiate into retinal neurons when Notch1 activity is diminished was not understood. To investigate this mechanism, we studied expression profiles of Notch1 receptor-bearing cells (Notch1^+^ RPCs) isolated from retinas at early and late developmental stages. Since expression of repressors and activators of neuronal differentiation should be present in Notch1^+^ RPCs, we conducted microarray analysis on a population of Notch1^+^ RPCs isolated from developing retinal tissue in order to create an expression profile of these progenitors. We suggested that the expression profile of Notch1^+^ RPCs should contain all genes involved in the Notch1 signaling cascade (Notch1 gene network), including activators which direct Notch1^+^ RPCs to differentiate into photoreceptors or ganglion cells when Notch1 activity is diminished. Our data indicate that Otx2 and Atoh7 function as such activators in Notch1^+^ RPCs.

## Results

### Notch1^+^ cells isolated from E14 and P0 retinas may represent two different populations of RPCs

**Fig 1:**
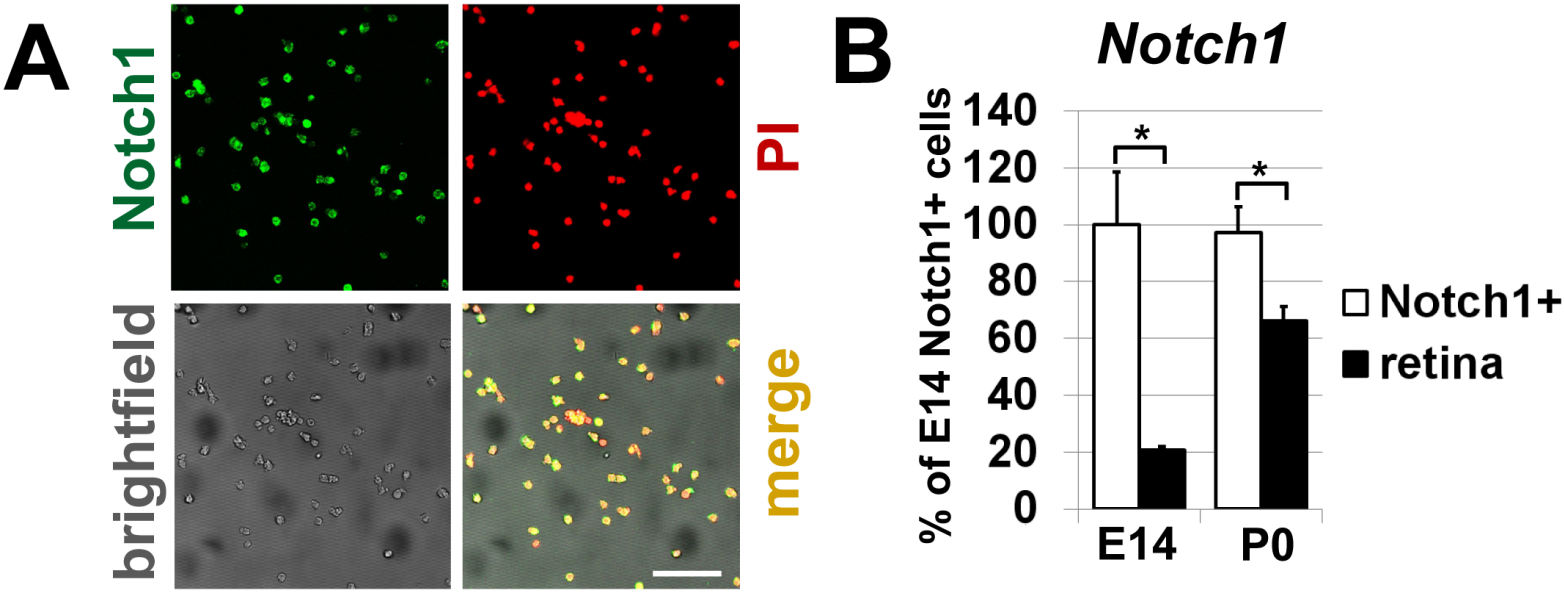
Immunomagnetic separation proved to be an effective method for positive selection of Notch1-specific cells from developing retinas. **A)** The purity of isolated cells was tested using anti-Notch1 antibody (green). Propidium iodide (PI, red) was used to visualize the nucleus of the cell. Bar is 50 μm. **B)** The levels of Notch1 expression in isolated cells were higher compared to expression of Notch1 in whole retinas. Gene expression was measured by qRT-PCR. Results are expressed as a percentage of the corresponding value in the Notch1^+^ cells isolated from embryonic day 14 developing retinas ± SEM (*P < 0.05).

In our study, we used retinas at embryonic day 14 (E14) and postnatal day 0 (P0) to evaluate changes in Notch1^+^ cells at early and late stages of retinal development. To isolate Notch1^+^ cells we employed an immunomagnetic cell separation approach using monoclonal biotin conjugated anti-Notch1 mouse antibodies and anti-biotin magnetic microbeads. This approach allowed us to isolate intact cells expressing the Notch1 protein at their surface. To confirm the purity, we tested the expression of Notch1 in the positively selected cells. Our data showed that 97–99% of these cells were immunopositive for Notch1 (Fig 1A). Using quantitative RT-PCR, we also evaluated the levels of Notch1 expression in isolated cells compared to expression of Notch1 in whole retinas. Our data indicated significantly higher expression of Notch1 in isolated cells compared to whole retina samples (Fig 1B). To demonstrate specificity of the isolated cells, we attempted to isolate cells from retinas using only the anti-biotin magnetic microbeads (negative control). No cells were detected after this procedure. Overall, immunomagnetic separation proved to be an effective technique for isolating Notch1^+^-specific cells from developing retinas.

**Fig 2:**
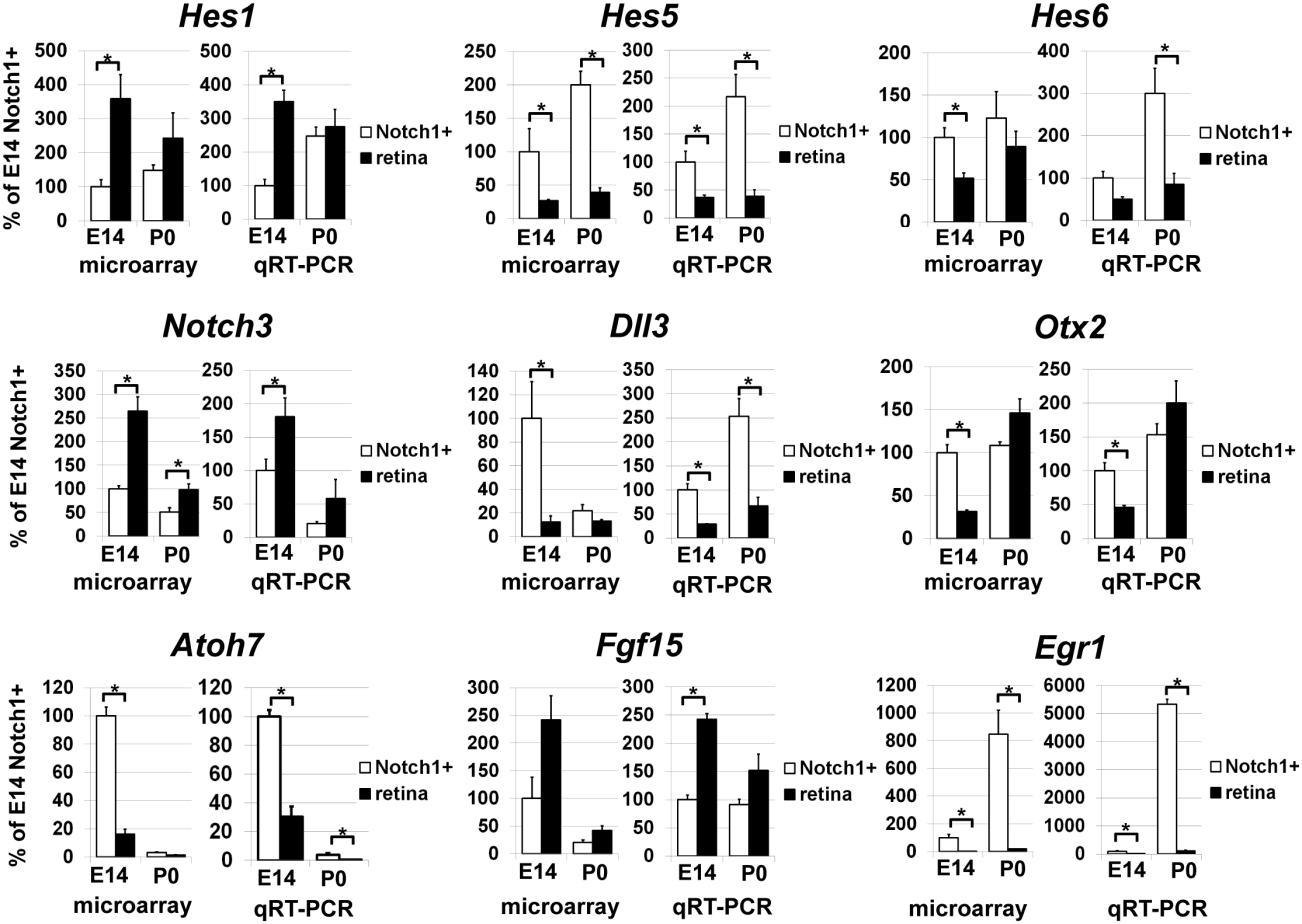
Differential gene expression was confirmed by qRT-PCR and demonstrated a consistent correlation with microarray data for a group of selected genes. For each gene, results are expressed as percentages ± SEM of the corresponding values in the Notch1^+^ cells isolated from E14 developing retinas (*P < 0.05).

To characterize Notch1^+^ cells isolated from retinas at E14 and P0, we studied gene expression in these cells using Mouse Exonic Evidence Based Oligonucleotide (MEEBO) microarrays, which included 38,083 genes and transcripts. To perform this experiment, RNA from isolated Notch1^+^ cells and whole retinas at E14 and P0 was extracted and used for microarray analysis. We processed individual samples that each contained 200,000–500,000 Notch1^+^ cells. A total of three independent biological replicates in the early stage (E14) of retinal development and four independent biological replicates in the late stage (P0) of retinal development were obtained for comparative profiling of isolated Notch1^+^ cells and whole retinas. The results of our microarray analysis indicated that a total of 6301 genes passed the quality control criteria and One-Way ANOVA test (*F* > *Fcrit*. = 3.708) to identify genes that are regulated differently between the four studied groups (E14 Notch1^+^, E14 Retina, P0 Notch1^+^, and P0 Retina—see S1 Table and Table 1). The changes in gene expression detected by microarrays were confirmed by quantitative RT-PCR for the group of genes that includes Notch1 (Figs 1 and 2, Table 1).

**Table 1:** Functional annotation for the selected genes revealed by One-Way ANOVA.

		signal intensity E14		signal intensity P0		
Gene	*F*	Notch1+	Retina	Notch1+	Retina	Function
*Atf4*	12.9	5686	2795	2367	3653	transcription factor
*Atoh7*	224.1	34524	5546	1037	485	transcription factor
*Brd7*	9.1	1910	634	1804	854	transcription factor
*Chd7*	15.1	17538	8562	8924	7359	transcription co-factor
*Dll1*	4.1	1365	528	2147	874	Notch signaling
*Dll3*	8.8	2815	348	620	371	Notch signaling
*Dll4*	2.5	540	320	636	205	Notch signaling
*Fgf15*	15.1	18004.2	43486.8	3703.9	7598	embryonic development
*Foxm1*	12.2	2418	1303	1519	1214	transcription factor
*Gadd45a*	25.6	22064	8374	4094	4019	maintains genomic stability
*Hes1*	3.9	1062	3807	1574	2573	transcription factor
*Hes5*	17.9	4074	832	6203	1213	transcription factor
*Notch1*	7.6	28131	9360	27451	15025	Notch signaling
*Notch3*	33.3	786	2081	401	770	Notch signaling
*Notch4*	6.8	238	169	103	146	Notch signaling
*Otx2*	19.8	25621	7996	27791	37445	transcription factor
*Rcor2*	18.5	4398	1841	2793	1584	transcription factor
*Sox11*	37.7	17303	12444	6527	5335	transcription factor
*Sox8*	11.4	100	301	1719	1532	transcription factor
*Taf5l*	7.6	3447	1372	1311	1885	transcription co-factor

**Fig 3:**
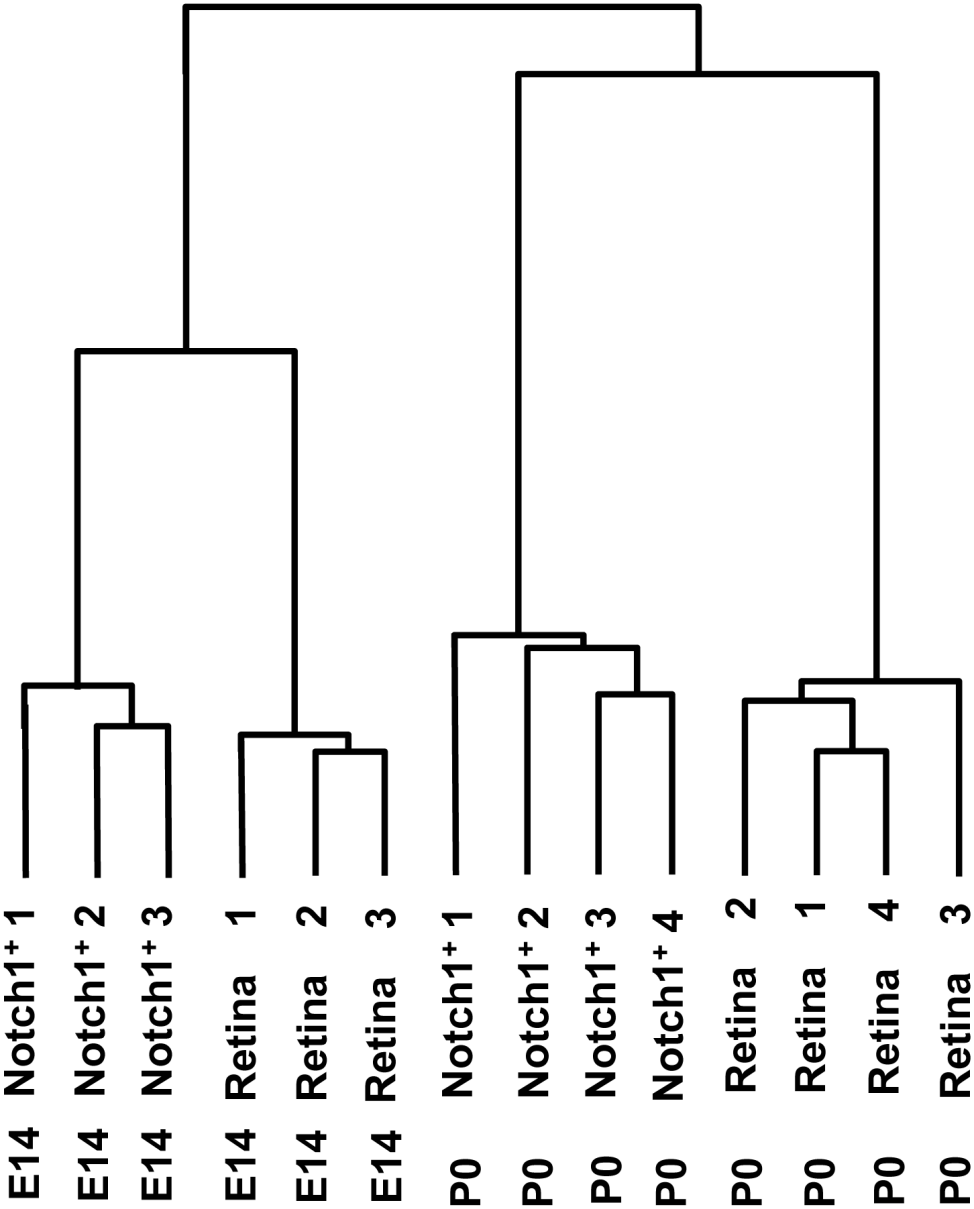
Hierarchical clustering of 6,301 differentially expressed genes showed that Notch1^+^ cells grouped near the same developmental stage retina cluster. Cluster analysis (hierarchical clustering) was performed using Gene Cluster 3.0 software.

Previously published data has demonstrated that there is a group of genes (predominantly transcription factors) that defines the competence as well as the specification and differentiation of RPCs into retinal neuronal cell types. For example, during embryonic retinal neurogenesis, Atoh7-expressing cells (Atoh7 lineage) preferentially differentiate into ganglion cells [8]. Meanwhile, Foxn4-expressing cells (Foxn4 lineage) give rise to amacrine and horizontal cells [8]. Otx2-expressing cells (Otx2 lineage) in early stages of retinal development differentiate predominantly into cone photoreceptors, while Otx2-expressing cells in late stages of retinal development differentiate predominantly into rod photoreceptors and bipolar cells [8, 28]. In light of these observations, we investigated whether Notch1^+^ cells represent a unique RPC lineage or if Notch1 signaling simply modulates differentiation potential in different RPC lineages. If Notch1^+^ cells do in fact represent a unique RPC lineage, they would not be expected to demonstrate significant changes in the expression of genes that regulate different specification or differentiation fates at E14 and P0. To answer this question of lineage, we examined changes in gene expression in Notch1^+^ cells isolated from E14 and P0 retinas. Using a Student's T-test and twofold cut-off level, we identified 1,185 genes that were preferentially expressed in the E14 Notch1^+^ cells and 740 genes preferentially expressed in the P0 Notch1^+^ cells (S2 Table). These findings suggested that Notch1^+^ cells at E14 and P0 may comprise two distinct cell populations. To verify this observation, we performed hierarchical clustering of differentially expressed genes. Our data indicated that gene expression profiles of E14 Notch1^+^ cells shared more similarities with gene expression profiles of E14 whole retinas than with the gene expression profiles of P0 Notch1^+^ cells. By the same token, expression profiles of P0 Notch1^+^ cells were more similar to expression profiles of P0 whole retinas than to expression profiles of E14 Notch1^+^ cells (Fig 3). Thus, Notch1 activity may not be restricted to one type of RPC, but may instead modulate the differentiation status of different types of RPCs, depending on their respective stages of retinal development.

### High levels of Otx2 and Atoh7 expression suggest that Notch1^+^ RPCs are predisposed to differentiate into photoreceptors and ganglion cells

As we noted in our “Introduction” section, the expression profile of Notch1^+^ RPCs should contain all genes involved in the Notch1 signaling cascade (Notch1 gene network), including those genes encoding repressors and activators of neuronal differentiation. To properly situate Notch1 signaling within a “coordinate system” of the complex gene network of retinal neurogenesis, we took Notch1^+^ cells at early and late stages of retinal development and tested if they expressed any markers characteristically expressed by RPCs, precursor cells, immature retinal neurons, and mature retinal neurons. The list of retinal cell type markers was obtained from LifeMap Discovery and The Stem Cell Research Database (http://discovery.lifemapsc.com). First, we found high expression levels of all key early RPC markers at E14 (S3 Table and Table 2). Many of them (such as Bmpr1a/ALK3, Cdc25b, Pax6, Ptch1, Six3) were more highly expressed in Notch1^+^ cells than in whole retina samples. However, expression of only two proteins (Notch1 and Hes5) was significantly up-regulated in Notch1^+^ cells compared to whole retina samples at E14. Meanwhile, expression of other RPC markers (including Bmpr1b/ALK6, Fgf15, Vsx2/Chx10, Hes1, Six6, and Sox2) was up-regulated in E14 whole retinas (S3 Table and Table 2). Importantly, Notch1 expression was higher compared to expression of other RPCs markers (Table 2). It should also be noted that Hes5 expression was higher than Hes1 expression (Table 2). We observed a similar expression pattern of late RPC markers in Notch1^+^ cells in late stages of retinal development (S3 Table and Table 2). We also analyzed expression of Notch1 ligands such as Dll1, Dll3, and Dll4. We found that all of these Notch1 ligands were expressed in Notch1^+^ cells at E14 and P0, and that their levels were higher in Notch1^+^ cells than in whole retina samples (Table 1). However, Notch1^+^ cells at E14 expressed higher levels of Dll3 than of Dll1 and Dll4, while Notch1^+^ cells at P0 expressed higher levels of Dll1 than of Dll3 or Dll4 (Table 1). These data suggest that the role of Dll3 in the Notch1 signaling cascade is most significant at E14, while Dll1 plays a more important role in Notch1 signaling at P0.

**Table 2:** The expression of retinal cell markers in Notch1^+^ progenitor cells and in whole retina samples at E14 and P0.

Gene	signal intensity E14		signal intensity P0		Notch1+: Retina E14		Notch1+: Retina P0		Retinal cell lineages	
	Notch1+	Retina	Notch1+	Retina	Ratio	p-value	Ratio	p-value	early	late
*Bmpr1a*	1953	1192	1771	756	1.63	0.289	2.34	0.007	RPC	
*Hes1*	1062	3807	1574	2573	0.27	0.025	0.61	0.262	RPC	RPC, MG
*Hes5*	4074	832	6203	1213	4.89	0.048	5.11	0.0002	RPC	RPC, MG
*Notch1*	28131	9360	27451	15025	3.01	0.007	1.82	0.044	RPC	RPC
*Pax6*	1858	1422	1276	1013	1.31	0.452	1.25	0.272	RPC, RGC, AC, HC	RPC, AC
*Atoh7*	34524	5546	1037	485	6.22	0.0003	2.13	0.052	RGC	
*Dlx1*	4193	1648	265	379	2.54	0.001	0.69	0.182	RGC	
*Dlx2*	3253	1700	868	982	1.91	0.106	0.88	0.231	RGC, AC, HC	
*Ebf2*	503	116	119	106	4.31	0.031	1.12	0.498	RGC	BC
*Ebf1*	315	3003	54	774	0.11	0.001	0.07	0.009	RGC, AC, BC	AC, BC
*Isl1*	824	4370	66	707	0.18	0.016	0.09	0.028	RGC, AC	BC
*Isl2*	331	271	59	565	1.22	0.657	0.10	0.006	RGC	
*Foxn4*	1068	640	1411	897	1.66	0.284	1.57	0.149	AC, HC	AC
*Bhlhe22*	1333	379	648	894	3.51	0.045	0.72	0.381	AC	BC
*Neurog2*	330	444	949	161	0.74	0.226	5.86	0.202	AC	BC
*Otx2*	25621	7996	27791	37445	3.21	0.001	0.74	0.065	Cone	Rod, BC
*Nr1d1*	199	50	313	102	3.93	0.0001	3.05	0.023		Rod
*Ascl1*	1460	902	3218	3570	1.61	0.152	0.90	0.741		Rod, BC
*Crx*	596	309	396	1590	1.93	0.188	0.24	0.052	Cone,	Rod, BC
*Six6*	5611	8672	3714	9137	0.64	0.042	0.41	0.0004	RPC	RPC
*Vsx2*	3783	6825	3306	6343	0.55	0.131	0.52	0.074	RPC	RPC, BC
*Sox2*	485	1112	264	873	0.43	0.048	0.30	0.051	RPC, AC	RPC, MG
*Sox9*	2285	3049	1601	3415	0.74	0.560	0.46	0.035	RPC	RPC, MG

(RPC—retinal progenitor cells, RGC—retinal ganglion cells, AC—amacrine cells, HC—horizontal cells, BC—bipolar cells, MG—Muller glia)

Next we analyzed the expression of markers of the precursor, immature, and mature stage of each cell type in Notch1^+^ cells at early and late stages of retinal development (S3 Table). In Notch1^+^ cells at E14 and P0, we observed insignificant or significantly down-regulated expression of many markers of immature and mature retinal neurons (S3 Table and Table 2). During early development (S3 Table) we found increased expression of Atoh7 (marker of ganglion precursor cells), Otx2 (marker of photoreceptor precursor cells), and Foxn4 (marker of amacrine and horizontal precursor cells) in Notch1^+^ cells at E14 compared to expression of these genes in whole retina samples (Table 2). However, levels of Atoh7 and Otx2 in Notch1^+^ cells at E14 were up to thirtyfold higher than levels of Foxn4 (Table 2). Importantly, Atoh7 and Otx2 were most highly expressed in Notch1^+^ cells at E14, with Atoh7 expression being even greater than Notch1 expression (Table 2). It should be noted that results of hybridization *in situ* from databases Eurexpress (http://www.eurexpress.org/ee/) and GenePaint.org (http://genepaint.org/Frameset.html) indicate that Notch1, Atoh7, and Otx2 expression at E14 spread throughout the neuroblast layer, but not into the ganglion cell layer. The results of our microarray analysis also demonstrated increased expression of Dlx1 and Dlx2 (immature ganglion cell markers) in Notch1^+^ cells, while expression of Isl1 and Pou4f2/Brn3b (markers of ganglion precursor cells) was significantly down-regulated (S3 Table and Table 2). We further observed increased expression of Crx and Rorb (markers of photoreceptor precursor cells) in Notch1^+^ cells at E14, though the increased expression of these genes was not statistically significant. However, at P0, expression of Crx and Rorb was higher in the whole retina samples than in Notch1^+^ cells. Thus, the gene expression profile of Notch1^+^ cells contains gene products that prevent neural differentiation (such as Notch1 and Hes5) and gene products that promote neuronal cell fate specification (such as Atoh7 and Otx2). In sum, the results of our study suggest that diminished Notch1 activity in Notch1^+^ RPCs may permit the highest and most prolonged expression of Atoh7 and Otx2. Because of this relationship, Notch1^+^ RPCs could be predisposed to differentiate into ganglion cells or photoreceptors when Notch1 activity is diminished. Finally, our results suggest that in the late stage of retinal neurogenesis Notch1^+^ RPCs may also differentiate into bipolar cells, because Otx2 additionally regulates bipolar cell development [8, 28].

As we outlined above, the expression profile of Notch1^+^ RPCs should contain all genes of the Notch1 gene network. However, results described in this and previous sections indicate that the Notch1 gene network at E14 and the Notch1 gene network at P0 may be two distinctly different networks. In accord with our data and the published literature, in the early stage of retinal development (E14), the Notch1 gene network may contain at least Notch1, Hes5, Dll3, Dll1, Atoh7, Dlx1, Dlx2, and Otx2, and regulates ganglion cell and photoreceptor differentiation. In the late stage of retinal development (P0), the Notch1 gene network may contain Notch1, Hes5, Dll1, and Otx2, and regulates photoreceptor and probably bipolar cell differentiation. To identify the most comprehensive list of genes of the E14 and the P0 Notch1 gene networks, we performed additional analyses of our microarray data. Expression of certain genes (such as housekeeping genes) should not differ greatly between Notch1^+^ cells and whole retina samples. Meanwhile, expression of genes specific for Notch1^+^ cells should be significantly higher in Notch1^+^ cells than in whole retina samples. Taking all this into account, we chose a group of genes (S4 Table and Table 3) that satisfied the following selection criteria: 1) expression in either E14 Notch1^+^ cells or P0 Notch1^+^ cells that was ten times greater than the threshold level; 2) expression in either E14 Notch1^+^ cells or P0 Notch1^+^ cells that was two times greater than expression levels in whole retina samples of the same developmental stage. We also paid special attention to genes encoding transcription factors. The list of mouse transcription factors (TFs) was obtained from the AnimalTFDB 2.0 database (http://bioinfo.life.hust.edu.cn/AnimalTFDB/). We used the milder conditions and selected TFs (S4 Table and Table 3) with statistically significantly higher expression in either E14 Notch1^+^ cells or P0 Notch1^+^ cells than in whole retinas of the same developmental stage. These methods allowed us to expand the list of putative members of the E14 and P0 Notch1 gene networks.

**Table 3:** The putative members of E14 Notch1 and P0 Notch1 gene networks.

E14 Notch1 gene network		P0 Notch1 gene network
E14	E14 and P0	P0
Dll3, Atoh7, Dlx1, Dlx2, Chd7, Sox11, Gadd45a, Eya2, Srf, Atf4, Rcor2, Taf5l, Pbrm1, Epc1, Tsc22d2, Limd1, Ebf2, Sox21, Lhx3, Mafk, Grb10, Rassf4, Sstr2, Fbxl12, Ube2s	Notch1, Dll1, Otx2, Hes5, Bhlhe22, Mybbp1a, Arl2bp, E2f2, Brpf1, Foxm1, Brd7, Zfp28, Olig2, Cecr2, Hnrpdl, Mbtps1, Dusp1, Odf2, Pitpnm1, Qscn6, Lrpb7	Gtl2, Gcnt1, Zfp13, Jun, Junb, Tle2, Nfia, Zcchc14, Cd72, Pacsin3, Slc25a28, Igf1r, Kifc5a

### Notch3 may play a less significant role in ganglion cell fate specification than previously thought

**Fig 4:**
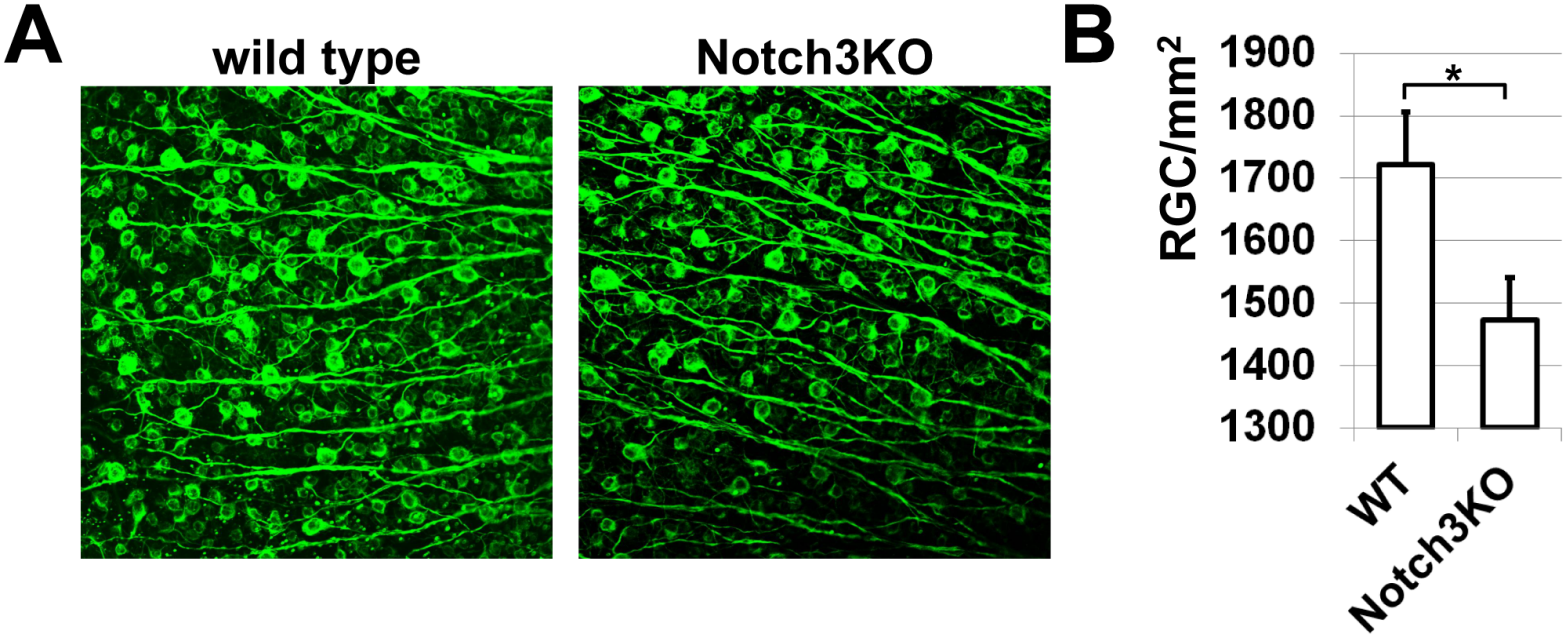
Notch3 deficiency results in reduced ganglion cell numbers in the retina. **A)** Confocal images of flat-mounted retinas from Notch3 knockout (Notch3KO) animals and wild type (WT) littermates were collected. RGCs were labeled with beta III Tubulin antibodies for counting. **B)** Numbers of RGCs were compared between Notch3KO and WT animals. Values are means ± SEM (*P < 0.05, n = 10 eyes).

The role of the Notch1 signaling cascade in photoreceptor differentiation was shown previously [21, 22, 24]. The critical role of both Notch1 and Notch3 signaling in ganglion cell fate specification was also demonstrated [8, 21]. However, these previous models described Notch3 as the primary regulator of ganglion cell development, with Notch1 playing a lesser, subsidiary role [21]. Our microarray data indicated that Notch3 was more highly expressed in whole retina samples than in Notch1^+^ cells at E14 and P0. We therefore reasoned that Notch3^+^ cells and Notch1^+^ cells may represent separate populations that may independently regulate ganglion cell differentiation. If Notch3, but not Notch1, significantly contributes to ganglion cell differentiation, then Notch3 deficiency should significantly affect the quantity of ganglion cells in the retina. To test this hypothesis, we used six-week-old Notch3 knockout (Notch3KO) animals and wild type littermates. The retinas of these animals were collected, and whole retina flat-mounts were stained for the retinal ganglion cell (RGC) marker beta III Tubulin in order to quantify the number of RGCs in the ganglion cell layer (Fig 4A). We found that retinas of wild type mice contained higher numbers of RGCs than retinas of Notch3KO mice (1,721±85 cells/mm^2^ vs. 1,474±67, *P*<0.05, Fig 4B). Therefore, retinas of wild type animals had 14% (*P* < 0.05) more RGCs than the retinas of Notch3KO mice. Since this number is relatively small and Notch1 contributes to ganglion cell (RGC) fate specification, we suggested that the Notch1 signaling cascade may play a more significant role in RGC development than the Notch3 signaling cascade.

## Discussion

The Notch family of receptors consists of four evolutionarily conserved, single-pass transmembrane receptor proteins (Notch1, Notch2, Notch3, and Notch4) that play a critical role in retinal neurogenesis [8–11, 21–25]. In the developing retina, members of the Notch family are involved in the maintenance of the RPC population, preventing neuronal differentiation until late stages [8–11, 21–25]. Meanwhile, inhibition of Notch activity facilitates differentiation of RPCs into photoreceptors and ganglion cells, depending on the stage of retinal development [8, 21–24]. The details of the RPC decision-making mechanism that guides differentiation into these retinal cell types, however, have never been clearly elucidated. To clarify this mechanism, we focused on the Notch1 receptor, whose role in retinal neurogenesis has been studied intensively [8, 21–24]. We separated Notch1 receptor-bearing cells (Notch1^+^ cells) from retinas at early and late developmental stages and studied the expression profiles of those cells. Our hierarchical clustering of differentially expressed genes suggested that Notch1^+^ cells at embryonic day 14 (E14, early stage of retinal development) may comprise a population of RPCs that are distinct from Notch1^+^ cells at postnatal day 0 (P0, late stage of retinal development). We observed significant expression of key transcription factors (Atoh7 and Otx2) in E14 Notch1^+^ cells and high expression of only Otx2 in P0 Notch1^+^ cells. These data suggested that E14 Notch1^+^ cells are already predisposed to differentiate into ganglion cells and photoreceptors, since at E14 Atoh7 facilitates differentiation of RPCs into ganglion cells and Otx2 regulates RPC fate decision into cone photoreceptors [8, 21, 22, 24]. Meanwhile, high levels of Otx2 expression in P0 Notch1^+^ cells may predispose them to differentiate into rod photoreceptors and probably bipolar cells, since Otx2 regulates differentiation into these cell types at P0 [8, 21, 22, 24, 28]. Because it was previously suggested that Notch3, but not Notch1, is primarily responsible for ganglion cell fate decision [21], we tested Notch3 expression in Notch1^+^ cells and evaluated whether ablation of Notch3 significantly affects ganglion cell numbers in the adult retina. Our data suggested that Notch3^+^ cells and Notch1^+^ cells may comprise distinct RPC populations. Notch3 knockout leads to reduced numbers of ganglion cells in adult retinas. But since the difference between wild type and Notch3KO retinas was only 14% (*P* < 0.05), we suggested that Notch1 signaling may play a more important role than Notch3 signaling in ganglion cell development at E14.

Notch signaling regulates the specification and differentiation of retinal cell types via a lateral inhibition mechanism, meaning that a cell fated to differentiate prevents differentiation of adjacent cells [8, 9, 11–14, 21–24]. To implement lateral inhibition, Notch ligands can trans-activate Notch in adjacent cells and induce expression of repressors of neuronal differentiation, such as Hes1 and Hes5 [8, 9, 11–14, 21–24]. Meanwhile, if Notch is not activated, absence of Hes1 and Hes5 expression permits expression of pro-neural transcription factors, which in turn induce neuronal-specific gene expression as well as expression of Notch ligands [8, 9, 11–14, 21–24]. Thus, a progenitor cell is predisposed to differentiate in the absence of Notch activity, but simultaneously prevents differentiation of adjacent cells due to its expression of Notch ligands. Our data indicate that Notch1^+^ cells express Notch ligands Dll1, Dll3, and Dll4. However, Dll3 and Dll1 were more highly expressed than Dll4. More specifically, Dll3 expression was higher than Dll1 at E14. Since our data indicated involvement of Notch1 signaling in the regulation of ganglion cell differentiation at E14 but not at P0, we can suggest that Dll3 may play an important role in the mechanism of ganglion cell differentiation. It should be noted, however, that Dll3 cannot *trans*-activate Notch signaling in neighboring cells [29, 30]. But Dll3 does inhibit (via *cis*-inhibition) Notch signaling when expressed by Notch receptor-bearing cells [29, 30]. We therefore speculate that Dll3 activity may affect ganglion cell fate during RPC differentiation. Meanwhile, Dll1 may be involved in photoreceptor differentiation (predominantly cones, as cones are the predominant phenotype at E14) during the early stage of retinal development, and may also facilitate rod photoreceptor and bipolar cell differentiation at the late developmental stage. Since Notch1 regulates neural/non-neural fate decisions based on Hes1 and Hes5 activity, we also paid attention to Hes1 and Hes5 expression in Notch1^+^ cells at different stages of retinal development. We detected high expression of both genes in Notch1^+^ cells at E14 and P0. However, expression of Hes5 was significantly higher than expression of Hes1. We also noted that while expression of Hes5 was up-regulated in Notch1^+^ cells compared whole retina samples at both E14 and P0, expression of Hes1 in Notch1^+^ cells was down-regulated compared to Hes1 expression in the whole retina samples. These data suggest that Hes5 may play a more important role than Hes1 in the Notch1 signaling cascade. Among the members of the Hes family, we would also like to pay attention to Hes6. Notch1^+^ cells exhibited significantly higher expression of Hes6 than of Hes1 and Hes5. It is known that Hes6 inhibits Notch/Hes1/Hes5 activity to promote neural differentiation [20, 31]. Taking into account that we did not detect statistically significant differences in Hes6 expression between Notch1^+^ cells and whole retina cell samples, we can suggest an important but not Notch1-specific role for Hes6 in retinal development.

The classical model of Notch-Dll lateral inhibition posited that progenitor cells with active Notch signaling cannot express pro-neural fate specification genes and therefore remain progenitor cells, while Dll-expressing cells activate pro-neural genes and become neurons, resulting in the “salt-and-pepper” histological pattern [14]. However, we now know that this classical model is reductive and obsolete. Kageyama first pointed out, using time-lapse imaging data, that the “salt-and-pepper” pattern of gene expression is actually a snapshot of oscillatory gene expression in adjacent cells [12–17]. These oscillations periodically activate Notch signaling between neighboring cells and allow expression of activators and repressors of pro-neuronal fate by the same cells. This reciprocal regulation is essential for the maintenance of a population of undifferentiated progenitor cells, allowing the developing tissue to accumulate a sufficient number of progenitors to differentiate and form normal tissue [12–17, 32]. Thus, we were not surprised when we observed a high expression of pro-neural genes (Atoh7, Otx2, Dll3, and Dll1) and inhibitors of neural differentiation (Notch1 and Hes5) in Notch1^+^ RPCs. Our results are also consistent with recent data that has demonstrated neural progenitor cells expressing pro-neural genes and Notch ligands at early developmental stages when these progenitors are not yet giving rise to any neurons [12–14, 18, 19, 26, 33–39]. The observation that RPCs express both repressors and activators of pro-neural fate specification had been published in the literature before the present study was undertaken [18–20]; our data merely corroborate those previously published observations. Combining our corroboratory observations with the oscillatory model of Notch-Dll lateral inhibition allows us to propose a putative Notch1^+^ RPC decision-making mechanism for differentiation into retinal neurons such as photoreceptors and ganglion cells. We suggest that Notch1 activation triggers Hes5 expression in Notch1^+^ RPCs, sparking an oscillatory expression pattern of activators (Atoh7 and Otx2) and repressors of pro-neural differentiation. These oscillations prevent RPC differentiation, while the stabilization of gene expression facilitates RPC differentiation. Since the level of Atoh7 and Otx2 expression was thirtyfold higher than the level of Foxn4 in Notch1^+^ cells at E14, we can expect that RPCs in which oscillations disappear will have consistently high Atoh7 and Otx2 expression compared to Foxn4 expression, and are therefore predisposed to differentiate into ganglion cells or photoreceptors at E14. At the same time, Notch1^+^ cells at P0 are predisposed to differentiate into Otx2-lineage neurons, since expression of Otx2 in these RPCs is significantly higher compared to Atoh7 and Foxn4 expression. Importantly, since oscillations of gene expression are the result of gene-gene interactions (gene network activity), our study allows us to identify putative members of the Notch1 gene network. It should be noted, however, that according to our data the Notch1 gene network at E14 differs from the Notch1 gene network at P0 (S4 Table and Table 3). Overall, the Notch1 gene network that contains Notch1, Dll3, Hes5, Atoh7, and Otx2 should regulate differentiation of ganglion cells and photoreceptors (predominantly cones) at the early stage of retinal development, while the Notch1 gene network containing Notch1, Dll1, Hes5, and Otx2 should affect photoreceptor (predominantly rods) and probably bipolar cell fate specification at the late stage of retinal development. The structure and dynamics of these gene networks are still not clear, and will require detailed future investigation to be elucidated. Our findings may serve as the foundation for such a study.

It was shown previously that Notch1 signaling regulates photoreceptor development at early and late stages of retinal development [21, 22, 24]. However, while a role of Notch1 in the regulation of ganglion cell fate was suggested, it was believed that Notch3 was primarily responsible for ganglion cell fate specification [21]. The best way to evaluate the respective roles of Notch1 and Notch3 in retinal development was to study the numbers of ganglion cells in wild type animals and gene knockouts. Unfortunately, the early embryonic lethality of Notch1-knockout mice and complexity of the Notch1 gene network (which can regulate development of many retinal cell types) created significant difficulties for evaluating the role of Notch1 in ganglion cell differentiation. Conversely, Notch3 knockout animals (Notch3KO) were viable and fertile, enabling us to use these animals in our study. We conjectured that the detected contribution of Notch3 in ganglion cell development would allow us to simultaneously evaluate the role of Notch1. Our expression data suggested that Notch3^+^ cells may comprise a population distinct from the Notch1^+^ cell population, and could therefore independently regulate ganglion cell differentiation. Results of ganglion cell counting in Notch3KO animals and wild type littermates revealed reduced numbers of ganglion cells in Notch3-deficient animals compared to wild type controls. This reduction was not extremely impressive (14%, *P* < 0.05), but was nevertheless statistically significant. Thus, we suggested that Notch3 may play some role in ganglion cell differentiation, but that its role may be less significant than expected. However, the reduced ganglion cell numbers that we observed in Notch3KO retinas suggested that Notch3 activates rather than inhibits ganglion cell development. This pro-differentiation effect of Notch3 has been previously described in the literature [40]. However, those observed effects can be explained by Notch3’s ability to inhibit neuronal differentiation. Our data indicated high expression in Notch1^+^ cells of Atoh7, which has been shown to prevent the differentiation of these cells into amacrine and horizontal cells [8, 9]. Congruently, our data demonstrated an at least thirtyfold lower expression of key amacrine and horizontal precursor cell markers (such as Foxn4, Ptf1a, Neurod1, Neurod4, and Dll4) in Notch1^+^ cells at E14 [8, 9]. In addition, we did not detect a statistically significant difference between the levels of these genes’ expression in Notch1^+^ cells and whole retina cell populations. Thus, since Notch3^+^ cells may represent a population distinct from the Notch1^+^ cell population, we speculate that Notch3 signaling could regulate (inhibit) differentiation of amacrine and horizontal cells, while Notch1 signaling could regulate the development of other neuronal types in retina. In this regard, reduced numbers of ganglion cells in Notch3KO retinas could mean preferential differentiation into amacrine and horizontal cells at the expense of ganglion cells. Regardless of whether Notch3 activates or inhibits ganglion cell development, the high Atoh7 expression in Notch1^+^ cells and low ganglion cell reduction in Notch3KO animals together suggest that the role of Notch1 signaling in ganglion cell fate specification should be important. Finally, we also evaluated expression of Notch2 and Notch4. Our data and the literature indicate that Notch2 levels in the developing retina are almost non-detectable [26, 27]. Meanwhile, we detected Notch4 expression in both Notch1^+^ cells and in whole retina samples, although Notch4 levels were significantly lower in comparison to Notch1 and Notch3. Notch4 is best known as a receptor primarily expressed in the vasculature and responsible for the regulation of vessel growth in the retina [25]. In total, these data suggest that the roles of Notch2 and Notch4 in retinal neurogenesis are less likely to be significant.

## Conclusions

The entire set of our data, the existing literature, and the “oscillatory” model of Notch-Dll lateral inhibition allow us to take a fresh look at Notch1 signaling in the developing retina. In general, Notch1 signaling is a gene network that contains progenitor markers (like Notch1 and Hes5) and pro-neural markers (like Atoh7 and Otx2). Expression of all of these genes should oscillate in RPCs, allowing these cells to maintain an undifferentiated state and proliferate. Nevertheless, RPCs are already predisposed to differentiate into particular retinal cell types when Notch1 inhibitory activity disappears. Our data suggest that at the early stage of retinal development the Notch1 gene network contains at least Notch1, Dll3, Hes5, Atoh7, and Otx2 (Table 4). This network may direct RPCs to differentiate into photoreceptors (cones) and ganglion cells. Meanwhile, the Notch1 gene network at the late stage of retinal development contains at least Notch1, Dll1, Hes5, and Otx2, and therefore may promote RPC differentiation into photoreceptors (rods) and probably bipolar cells. We cannot rule out that, at different stages of retinal development, the Notch1 gene network can contain different pro-neural markers that may direct RPC differentiation into distinct neuronal cell types (for example, Foxn4 lineage). The reconstruction of such gene networks and the crosstalk between them during retinal development could significantly change our views on the mechanisms of retinal neurogenesis.

**Table 4:** List of RT-PCR primers.

Gene	Oligonucleotides	
***Notch1***	Forward	TGTTGTGCTCCTGAAGAACG
	Reverse	GTGGGAGACAGAGTGGGTGT
***Notch3***	Forward	CTGTGCTACAGCCGTGTGTT
	Reverse	ATTCACACACCGACCCAAAT
***Hes1***	Forward	ACACCGGACAAACCAAAGAC
	Reverse	GTCACCTCGTTCATGCACTC
***Hes5***	Forward	CAAGGAGAAAAACCGACTGC
	Reverse	GTGCAGGGTCAGGAACTGTA
***Hes6***	Forward	CGGATCAACGAGAGTCTTCAG
	Reverse	GGCATGGATTCTAGCAGGTG
***Atoh7***	Forward	ATGAAGTCGGCCTGCAAAC
	Reverse	GGTGAGCGCGATGATGTAG
***Otx2***	Forward	GGGCTGAGTCTGACCACTTC
	Reverse	GGCCTCACTTTGTTCTGACC
***Dll3***	Forward	GGTCCCTGTCTCCACCAGTA
	Reverse	TGGGCAATGACAGACATAGG
***Fgf15***	Forward	GCTGGTCCCTATGTCTCCAA
	Reverse	GGAGATGGTGCTTCATGGAT
***Egr1***	Forward	TCACCCACCATGGACAACTA
	Reverse	GGGATAACTCGTCTCCACCA
***Actb***	Forward	CACCCTGTGCTGCTCACC
	Reverse	GCACGATTTCCCTCTCAG

## Material and Methods

### Animals

All experiments were performed in compliance with the National Institutes of Health (NIH) Guide for the Care and Use of Laboratory Animals and the Association for Research in Vision and Ophthalmology (ARVO) statement for use of animals in ophthalmic and vision research. The protocol was approved by the Institutional Animal Care and Use Committee (IACUC) of the University of Miami (Permit Number: 12–068). Notch3 knockout animals (stock number 023807) and C57BL/6 J (stock number 000664) mice were obtained from Jackson Laboratory (Bar Harbor, Maine, United States). Mice were housed under standard conditions of temperature and humidity, with a 12-hour light to dark cycle and free access to food and water.

### Isolation of Notch1^+^ cells

Eyes (n ≥ 20/preparation) from embryonic day 14 (E14) and postnatal day 0 (P0) C57BL/6 mice were enucleated, and the retina of each was dissected free and stored on ice in a CO2-independent medium (Invitrogen, Carlsbad, CA). Upon completion of the dissection, the CO2-independent medium was removed and replaced with the papain solution (8 U/mL of papain [Worthington Biochemicals, Lakewood, NJ] and 124 U/ml of DNAse I [Sigma-Aldrich, St. Louis, MO] in DPBS [Life Technologies, Grand Island, NY], with pH adjusted to 7.4). The retinas were incubated in the papain solution for 20 minutes at 37°C. After incubating the retinas, the old papain solution was removed and the retinas were rinsed with low ovomucoid solution (1.5 mg/ml of trypsin inhibitor ovomucoid [Worthington Biochemicals, Lakewood, NJ], 1.5 mg/ml of BSA [Sigma-Aldrich, St. Louis, MO], and 124 U/ml of DNAse I in DPBS, with pH adjusted to 7.4). The pieces were allowed to settle, and the rinse solution was removed. The tissue was then triturated sequentially with a 1 ml pipette in the low ovomucoid solution until the retinas were completely broken up. The retinal cell suspension resulting from the trituration was spun at 300 g for 10 minutes to separate the retinal cells from the low ovomucoid solution. The supernatant was discarded, and the cells were then resuspended in high ovomucoid solution (10 mg/ml of trypsin inhibitor ovomucoid and 10 mg/ml of BSA in DPBS, pH was adjusted to 7.4). The retinal cell suspension was spun at 300 g for 10 minutes, the supernatant was discarded, and the cells were resuspended in MACS buffer containing 0.5% BSA and 2 mM EDTA in PBS (pH 7.2). Viability was assessed by trypan blue exclusion, and was found to be greater than 97%.

To isolate Notch1^+^ cells, we used monoclonal biotin conjugated anti-Notch1 mouse antibodies (130-096-557, Miltenyi Biotec, Auburn, CA) and followed a protocol for immunomagnetic separation provided by Miltenyi Biotec (Auburn, CA). Briefly, the retinal cell number was first determined. The retinal cell suspension was then spun at 300g for 10 minutes, resuspended at up to 10^7^ nucleated cells per 100 μl of MACS buffer and 10 μl of the anti-Notch1 antibody, and incubated for 10 minutes in the refrigerator (2-8°C) in the dark. The retinal cell suspension was washed two times by adding MACS buffer and centrifuged at 300g for 10 minutes, resuspended at up to 10^7^ nucleated cells per 80 μl of MACS buffer and 20 μl of the anti-biotin microbeads (130-090-485, Miltenyi Biotec, Auburn, CA), then incubated for 15 minutes in the refrigerator in the dark. The cells were washed two times as above, resuspended at up to 10^8^ nucleated cells per 500 μl of MACS buffer, and used for magnetic separation of the Notch1^+^ cells on a pre-equilibrated column in the presence of a magnetic field according to the manufacturer's protocol (Miltenyi Biotech, Auburn, CA).

### RNA extraction, probe preparation, and array hybridization

RNA samples were extracted from whole retina and Notch1^+^ cells using the Absolutely RNA Nanoprep kit (Agilent Technologies, Santa Clara, CA). RNA samples were sent to Ocean Ridge Biosciences (ORB, Palm Beach Gardens, FL) for analysis using mouse exonic evidence-based oligonucleotide (MEEBO) microarrays (Lot 20918). MEEBO microarrays were printed by Microarrays Inc. (Huntsville, AL) and contained 38,083 70-mer oligonucleotide probes complementary to constitutive exons of most mouse genes, as well as alternatively spliced exons and control sequences. For more information on the MEEBO oligonucleotide set please refer to http://alizadehlab.stanford.edu/heebo.html. Biotin-labeled complementary RNA was made from total RNA according to Van Gelder’s protocol [41]. Biotinylated complementary RNA samples were fragmented, diluted in a formamide-containing hybridization buffer, and loaded on to the Mouse Exonic Evidence Based Oligonucleotide (MEEBO) microarray slides enclosed in custom hybridization chambers (for more information on the MEEBO oligonucleotide set please refer to http://alizadehlab.stanford.edu/). The slides were hybridized for 16–18 hours in a Model 400 hybridization oven (Scigene, Sunnyvale, CA). After hybridization, the microarray slides were washed under stringent conditions, stained with Streptavidin-Alexa-647 (Invitrogen, Carlsbad, CA), and scanned using an Axon GenePix 4000B scanner (Molecular Devices, Sunnyvale, CA).

### Microarray data analysis

Spot intensities for each probe were calculated by subtracting median local background from median local foreground for each spot. The spot intensities were then normalized. After removing data for low quality spots, the mouse probes’ intensities were filtered to identify all probes with intensity above a normalized threshold. For statistical analysis, microarray data were examined for differences by One-way ANOVA or Student's t-test. Values of *P* < 0.05 were designated as statistically significant. The data files have been uploaded in the NCBI Gene Expression Omnibus (GEO, http://www.ncbi.nlm.nih.gov/geo/) and are available through GEO series accession number GSE65977. Hierarchical cluster analysis (hierarchical clustering) was performed using Gene Cluster 3.0. To this end, Log2-transformed, significant (F > F crit = 3.708) mouse probes were loaded into Gene Cluster 3.0. The data were adjusted by centering genes (median). Clustering was performed using centered correlation as a distance measure and average linkage as the method. TreeView Software was used to visualize clustering results.

### Quantitative RT-PCR analysis

Quantitative RT-PCR analysis was performed as described previously [42, 43] using gene-specific primers (Table 4). Briefly, RNA samples were extracted from whole retinas and Notch1^+^ cells using the Absolutely RNA Nanoprep kit (Agilent Technologies, Santa Clara, CA) and reverse transcribed with Reverse Transcription System (Promega, Madison, WI) to synthesize cDNA. Quantitative PCR was then performed (Rotor-Gene Q, Qiagen, Valencia, CA) using a kit (SYBR GREEN PCR MasterMix; Qiagen, Valencia, CA). Relative expression was calculated by comparison with a standard curve following normalization to expression of the housekeeping gene β-actin (*Actb*), chosen as a control. Data are presented as average ± SEM. Quantitative RT-PCR measurements were analyzed with Student's t-test. Values of P < 0.05 were designated as statistically significant.

### Immunocytochemistry

Notch1^+^ cells were placed on cover slips, fixed after 30 minutes in 4% PF, and blocked with 5% normal donkey serum with 0.15% Tween-20 in PBS at pH 7.4. Cells were then incubated with Notch1 primary antibody (130-101-868, Miltenyi Biotec Inc., San Diego, CA) followed by species-specific secondary fluorescent antibodies (Invitrogen, Carlsbad, CA). Negative controls were incubated with secondary antibodies only. Imaging was performed with a confocal microscope (Leica TSL AOBS SP5; Leica Microsystems). Propidium iodide (PI) was used to visualize the nucleus of the cell. Individual cover slips were sampled randomly to collect a total of 10 images using a 20X objective lens. The Notch1 positive cells and PI positive cells (the total number of cells) were counted using ImageJ software. The percentage of Notch1 positive cells relative to the total number of cells was determined.

### Immunohistochemistry for beta III tubulin and counting of ganglion cell layer (GCL) neurons

Immunohistochemistry for beta III tubulin was performed as described previously [44]. Briefly, eyes were enucleated upon euthanasia and fixed in a 4% PF. The retinas were removed after 1 hour and cryoprotected overnight in 30% sucrose. The following day, the retinas underwent 3 freeze–thaw cycles, were rinsed in 0.1 M Tris buffer, blocked (5% donkey serum and 0.1% Triton X-100 in 0.1 M Tris buffer) for 1 hour, and were then incubated overnight with beta III Tubulin antibody (1:250; Covance, Denver, PA). After rinsing in 0.1 M Tris buffer, the retinas were flatmounted and coverslipped. Beta III Tubulin-positive GCL neurons were imaged by confocal microscopy. Individual retinas were sampled randomly to collect a total of 20 images from four retinal quadrants. Beta III Tubulin-positive GCL neurons were counted using ImageJ software.

## Supporting Information

S1 Table The results of microarray analysis revealed a total of 6,301 genes, which passed the quality control criteria and One-Way ANOVA test (*F* > *Fcrit*. = 3.708). (XLSX)

S2 Table Our findings suggested that Notch1^+^ cells at E14 and P0 may comprise two distinct cell populations. We identified 1,185 genes that were preferentially expressed in the E14 Notch1^+^ cells and 740 genes preferentially expressed in the P0 Notch1^+^ cells using a Student's T-test and twofold cut-off level.
(XLSX)

S3 Table The expression of retinal cell markers in Notch1^+^ progenitor cells and in whole retina samples was detected at E14 and P0. (XLSX)

S4 Table The list of putative members of the E14 and P0 Notch1 gene networks was identified. We chose a group of genes that satisfied the following selection criteria: 1) expression in either E14 Notch1^+^ cells or P0 Notch1^+^ cells that was ten times greater than the threshold level; 2) expression in either E14 Notch1^+^ cells or P0 Notch1^+^ cells that was two times greater than expression levels in whole retina samples of the same developmental stage. We used the milder conditions and selected transcription factors (TFs) with statistically significantly higher expression in either E14 Notch1^+^ cells or P0 Notch1^+^ cells than in whole retinas of the same developmental stage.
(XLSX)
